# Single-cell analysis of skin immune cells reveals an Angptl4-ifi20b axis that regulates monocyte differentiation during wound healing

**DOI:** 10.1038/s41419-022-04638-7

**Published:** 2022-02-24

**Authors:** Wei Kiat Jonathan Wee, Zun Siong Low, Chin Kiat Ooi, Benjamin Patrana Henategala, Zhi Guang Ridley Lim, Yun Sheng Yip, Marcus Ivan Gerard Vos, William Wei Ren Tan, Hong Sheng Cheng, Nguan Soon Tan

**Affiliations:** 1 Lee Kong Chian School of Medicine, Nanyang Technological University Singapore, 11 Mandalay Road, Singapore, 308232 Singapore; 2 School of Biological Sciences, Nanyang Technological University Singapore, 60 Nanyang Drive, Singapore, 637551 Singapore

**Keywords:** Acute inflammation, Acute inflammation

## Abstract

The persistent inflammatory response at the wound site is a cardinal feature of nonhealing wounds. Prolonged neutrophil presence in the wound site due to failed clearance by reduced monocyte-derived macrophages delays the transition from the inflammatory to the proliferative phase of wound healing. Angiopoietin-like 4 protein (Angptl4) is a matricellular protein that has been implicated in many inflammatory diseases. However, its precise role in the immune cell response during wound healing remains unclear. Therefore, we performed flow cytometry and single-cell RNA sequencing to examine the immune cell landscape of excisional wounds from Angptl4^+/+^ and Angptl4^−/−^ mice. Chemotactic immune cell recruitment and infiltration were not compromised due to Angptl4 deficiency. However, as wound healing progresses, Angptl4^−/−^ wounds have a prolonged neutrophil presence and fewer monocyte-derived macrophages than Angptl4^+/+^ and Angptl4^LysM−/−^ wounds. The underlying mechanism involves a novel Angptl4-interferon activated gene 202B (ifi202b) axis that regulates monocyte differentiation to macrophages, coordinating neutrophil removal and inflammation resolution. An unbiased kinase inhibitor screen revealed an Angptl4-mediated kinome signaling network involving S6K, JAK, and CDK, among others, that modulates the expression of ifi202b. Silencing ifi202b in Angptl4^−/−^ monocytes, whose endogenous expression was elevated, rescued the impaired monocyte-to-macrophage transition in the in vitro reconstituted wound microenvironment using wound exudate. GSEA and IPA functional analyses revealed that ifi202b-associated canonical pathways and functions involved in the inflammatory response and monocyte cell fate were enriched. Together, we identified ifi202b as a key gatekeeper of monocyte differentiation. By modulating ifi202b expression, Angptl4 orchestrates the inflammatory state, innate immune landscape, and wound healing process.

## Introduction

Poor healing wounds are a silent epidemic affecting a large fraction of the world population, often overshadowed by comorbid conditions such as diabetes [1]. The biomedical and socioeconomic burdens are expected to worsen as the population ages. Patients with poor healing wounds frequently find themselves forced to choose between their work commitments and compliance with medical management. Others may be permanently impaired from performing their occupational job. Thus, much attention and resources are needed to understand the biological mechanisms underlying cutaneous wound complications.

Normal wound healing consists of a finely tuned pattern of integrated biological events that include hemostasis, inflammation, proliferative, and maturation phases to re-establish a new epithelial barrier [2]. Inflammation plays both positive and negative roles in wound healing – the level and length of inflammation dictate the healing time and quality of the repair. Neutrophils are among the earliest immune cells recruited to the wound site [3]. While neutrophils play important roles in the early inflammatory phase of the wound, their timely apoptosis, followed by uptake by phagocytic macrophages, is a key event necessary for inactivation and departure from the inflammatory phase of wound repair [4]. However, chronic wounds are in pathologic inflammation due to a postponed, incomplete, or uncoordinated healing process [5]. Cell–cell and cell–matrix communication, which coordinates the essential and complex inflammatory response during normal wound healing, is disrupted in chronic wounds. This synchrony is mediated by the action of central players, such as chemokines and matricellular proteins [6]. Matricellular proteins can associate with diverse proteins of the extracellular matrix, bridging them with cognate cell surface receptors and contributing to paracrine communication during wound healing [7]. However, there is still much to learn about the dynamics of cell–matrix communication that modulate inflammation in acute skin wounds and how it becomes deranged to generate chronic wounds.

Angiopoietin-like 4 protein (Angptl4) is a secreted matricellular protein that undergoes proteolytic cleavage, releasing an N-terminal coiled-coil domain (nAngptl4) and a C-terminal fibrinogen-like domain (cAngptl4). nAngptl4 binds to lipoprotein lipase and inhibits its activity to regulate peripheral triglyceride metabolism [8]. cAngptl4 interacts with specific integrins, cadherins, and ECM proteins to enhance their signaling [9, 10]. Angptl4 has been implicated in modulating immune responses in various inflammatory diseases. Hematopoietic Angptl4 deficiency in hyperlipidemic mice causes leukocytosis [11]. Angptl4-deficient (Angptl4^−/−^) mice were shown to have large numbers of macrophages in the spleen, and Angptl4^−/−^ macrophages produced large amounts of TNF-α and inducible nitric oxide synthase due to abnormal fatty acid metabolism [12]. It was also shown that dietary fatty acids activate PPARβ/δ signaling in the bone marrow to negatively regulate mobilization, in part via Angptl4 production. The treatment of wild-type (Angptl4^+/+^) mice with anti-cAngptl4 antibody enhanced mobilization [13]. Despite its involvement in many inflammatory diseases, the precise role of Angptl4 in immune cells and responses remains unclear.

Angptl4 participates in many events that are important for proper wound healing. Upon wound injury, hyperproliferative wound keratinocytes secrete a large amount of cAngptl4 that acts as a coordinating conduit with other wound cell types [14]. cAngptl4 facilitates wound angiogenesis, accelerates keratinocyte and fibroblast migration, and reduces scar collagen deposition [15]. Wound healing in Angptl4^−/−^ mice is delayed and shares many similarities with diabetic wounds. Wounds are associated with stalled inflammation, poor wound-related angiogenesis, and ruined ECM. Indeed, human and mouse diabetic wounds produce little cAngptl4 compared with nondiabetic wounds. The topical application of recombinant cAngptl4 accelerates wound closure in diabetic mice [16]. However, how Angptl4 regulates cell-matrix communication to affect the immune cell response to injury is unknown. A better understanding will contribute to translatable therapies for chronic wounds and many other inflammatory diseases.

This study examines the immune cell landscape of excisional wounds from wild-type (Angptl4^+/+^) and Angptl4^−/−^ mice. We uncovered that Angptl4 deficiency in the wound site reduces monocyte-derived macrophages and prolongs the inflammatory phase of wound healing. In addition, Angptl4 regulates the expression of interferon-activated gene 202B (ifi202b), which affects gene networks involved in monocyte cell fate.

## Results

### Angptl4 deficiency portends prolonged inflammation in wounds

We first examined the temporal changes in immune subpopulations using a multicolor flow cytometry panel for CD11b (myeloid cell marker), Ly6G (neutrophil marker), Ly6C (mouse monocyte marker), and MHC-II (macrophage marker). Figure S1A shows representative plots outlining the gating strategy. Four subpopulations of monocytes and macrophages were observed in our excisional wound model – namely, Ly6C^hi^MHC-II^-ve^ inflammatory monocytes, Ly6C^int^MHC-II^-ve^ monocytes, and Ly6C^+ve^MHC-II^+ve^ and Ly6C^-ve^MHC-II^+ve^ monocyte-derived macrophages. Next, we examined the changes in these immune cell subpopulations during wound healing in Angptl4^+/+^ and Angptl4^−/−^ mice. At 1 day postwounding (dpw), no significant difference in infiltrated neutrophils, inflammatory monocytes, and Ly6C^+ve^MHC-II^+ve^ monocyte-derived macrophages was observed between Angptl4^+/+^ and Angptl4^−/−^ wounds (Table 1, Fig. S1B). Interestingly, Angptl4^−/−^ wounds had a higher percentage of infiltrated monocytes at 1 to 3 dpw but a reduced percentage of mature Ly6C^-ve^MHC-II^+ve^ macrophages at 1 to 5 dpw compared with Angptl4^+/+^ wounds. Consistent with a role for macrophages in the clearance of neutrophils, Angptl4^−/−^ wounds had prolonged neutrophil persistence at 5- to 7-dpw. There were fewer Ly6C^-ve^MHC-II^+ve^ mature monocyte-derived macrophages in Angptl4^−/−^ than Angptl4^+/+^ wounds across all examined time timepoints (Table 1). Following the peak of Ly6C^+ve^MHC-II^+ve^ and Ly6C^-ve^MHC-II^+ve^ monocyte-derived macrophages at 3 dpw, their percentage decreased from 5- to 7-dpw in Angptl4^+/+^ wounds (59−77%). In contrast, their percentage increased in Angptl4^−/−^ wounds (28–50%) (Table 1). Cytokine profiling of early wound exudates at 8 h and 1 dpw from Angptl4^+/+^ and Angptl4^−/−^ wounds showed no significant difference in the examined 31 cytokines (Fig. S2A). An in vitro chemotaxis assay using bone marrow-derived monocytes (BMDMs) from Angptl4^+/+^ and Angptl4^−/−^ mice also showed no difference in invasiveness, suggesting that chemotactic immune cell recruitment and infiltration were not compromised due to Angptl4 deficiency (Fig. S2B). Finally, we also examined neutrophils and macrophages in wounds of Angptl4^LysM−/−^ myeloid cell-specific Angptl4 deletion. Similar numbers of neutrophils and Ly6C^-ve^MHC-II^+ve^ mature macrophages were found in Angptl4^LysM−/−^ and Angptl4^f/f^ wounds (Fig. S2C). Hence, Angptl4 deficiency affects cell-matrix interactions that potentially regulate monocyte to macrophage differentiation and portends prolonged inflammation during wound healing.

**Table 1:** Percentage of immune cell subpopulations in Angptl4^+/+^ and Angptl4^−/−^ wounds as determined by flow cytometry.

	Neutrophils		Ly6C^hi^MHC-II^-ve^ Inflammatory monocytes		Ly6C^int^MHC-II^-ve^ Monocytes		Ly6C^+ve^MHC-II^+ve^ Differentiating macrophages		Ly6C^-ve^MHC-II^+ve^ Macrophages	
dpw	Angptl4^+/+^	Angptl4^−/−^	Angptl4^+/+^	Angptl4^−/−^	Angptl4^+/+^	Angptl4^−/−^	Angptl4^+/+^	Angptl4^−/−^	Angptl4^+/+^	Angptl4^−/−^
1	31.1 ± 0.8	31.6 ± 0.7	7.8 ± 1.5	6.9 ± 0.6	1.8 ± 0.2	2.8 ± 0.1*	0.78 ± 0.1	0.73 ± 0.1	0.21 ± 0.02	0.08 ± 0.01*
3	24.5 ± 0.5	29.2 ± 0.3	0.8 ± 0.4	0.9 ± 0.6	0.9 ± 0.1	1.8 ± 0.3*	0.67 ± 0.03	0.71 ± 0.2	0.47 ± 0.04	0.29 ± 0.03*
5	4.9 ± 0.7	17.9 ± 0.3 *	0.7 ± 0.2	0.6 ± 0.2	0.23 ± 0.06	0.43 ± 0.03*	0.27 ± 0.2	0.32 ± 0.1	0.13 ± 0.05	0.06 ± 0.02*
7	0.8 ± 0.3	4.3 ± 0.7 *	0.3 ± 0.1	0.5 ± 0.3	0.07 ± 0.02	0.09 ± 0.01	0.11 ± 0.04	0.41 ± 0.1*	0.03 ± 0.01	0.09 ± 0.008*

Data are from *n* = 4 independent experiments with 3 mice at each time point. *p value < 0.05.

### Angptl4 deficiency results in impaired monocyte differentiation during wound healing

Guided by our flow cytometry findings, single-cell RNA sequencing (scRNA-seq) was performed on sorted CD45^+^ cells from wound biopsies of Angptl4^+/+^ and Angptl4^−/−^ mice at 1 and 5 dpw. Cell sorting procedures were optimized, and 19,579 sequenced cells that met the quality control metrics were analyzed (Fig. S3). Six major immune cell clusters were identified from the aggregated scRNA-seq data (Fig. S4A). Probing the top 25 significantly enriched genes from each cluster against the ImmGen database [17] showed a close correlation with datasets from specific immune cell types, confirming the accurate annotation of the cell clusters (Fig. S4B). Thus, acute excisional skin wounds comprise mostly myeloid cells (neutrophils, monocytes, macrophages, and dendritic cells), with minor NK and T subpopulations.

**Fig. 1:**
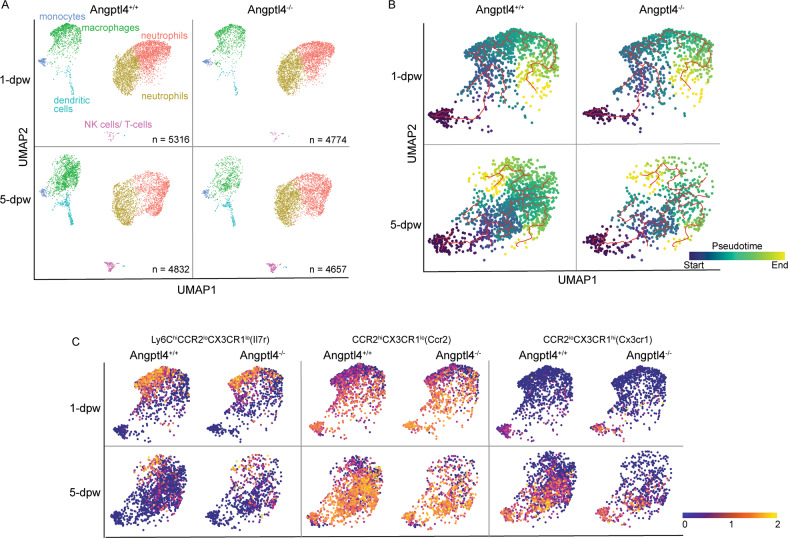
Single-cell immune landscape during mouse skin excisional wound healing. **A** Individual UMAP dimensionality reduction plots of sequenced CD45^+^ immune cells from Angptl4^+/+^ and Angptl4^−/−^ wounds at 1 and 5 days post-wounding (dpw). **B** UMAP dimension reduction plots showing cells from individual scRNA-seq samples ordered by pseudotime (color scale). Monocytes were marked as the start of pseudotime. Calculated cell trajectories are shown as red lines. **C** The distribution of cells expressing Il7r, Ccr2 and Cx3cr1 during the recovery of Angptl4^+/+^ and Angptl4^−/−^wounds.

Two clusters of neutrophils were identified from cluster annotation and analysis of the differentially expressed genes (DEGs) (Fig. S5A). Cluster 0 neutrophils expressed many protumorigenic factors, such as Cxcr2, TGF-β1, Mmp9, and Lrg1. Cluster 1 neutrophils expressed various proinflammatory and proangiogenic factors, such as VEGF-A, TNF-α, and Ccl3 (Fig.S5A). In Angptl4^+/+^ wounds, infiltrated neutrophils (clusters 0 and 1) decreased by 17.5% from 1 to 5 dpw. In contrast, the decrease was only 2.3% in Angptl4^−/−^ wounds, consistent with neutrophil persistence (Fig. 1A, Table 2).

**Table 2:** **I**mmune cell subpopulations in Angptl4^+/+^ and Angptl4^−/−^ wounds by scRNA-seq.

	% of infiltrated CD45^+^ cells	1-dpw		5-dpw	
Cluster	Cell type	Angptl4^+/+^	Angptl4^−/−^	Angptl4^+/+^	Angptl4^−/−^
0	neutrophils	39.95	42.73	30.11	39.49
1	neutrophils	33.13	34.67	25.50	35.60
2	macrophages	21.61	18.06	30.69	13.64
3	dendritic cells	1.90	1.76	7.16	4.49
4	monocytes	2.73	2.09	2.30	2.30
5	NK cells/T cells	0.68	0.69	4.22	3.97

The number of cells in each cluster was expressed as a percentage of the total number of CD45^+^ cells for each sample.

The monocyte cluster was highly enriched in Ly6c2 expression, whereas monocyte-derived macrophages expressed high levels of MHC-II (H2-Aa) and Adgre1, or F4/80, a common macrophage marker. Moderate to low expression of Ly6c2 was enriched in the macrophage cluster, likely representing monocytes differentiating into macrophages (Fig. S5B). Very few cells expressed Cd207 and Cd169, biomarkers of Langerhans cells and dermal resident macrophages at 1 dpw, respectively [18] (Fig. S5C). No difference in infiltrated monocytes was observed between the two genotypes. The percentage of monocyte-derived macrophages increased by 9.1% in Angptl4^+/+^ wounds, whereas the percentage decreased in Angptl4^−/−^ wounds from 1 to 5 dpw (Table 2). Trajectory analysis revealed that monocytes have two main developmental branches (Fig. 1B), indicative of early and late wound immune responses [19]. Angptl4 deficiency resulted in reduced numbers of Ly6C^hi^CCR2^lo^CX3CR1^lo^ (Il7r > 1) inflammatory monocytes, CCR2^hi^CX3CR1^lo^ (Ccr2, Adgre1 > 1 & Ly6c2 < 1) and CCR2^lo^CX3CR1^hi^ (Cx3cr1, Adgre1 > 1 & Ly6c2 < 1) monocyte-derived macrophages, with the most significant deficit in the number (>50%) of monocyte-derived macrophages (Fig. 1C, Table 3). There was no significant difference in dendritic cells at 1 dpw between the two genotypes; however, the percentage was lower at 5 dpw in Angptl4^−/−^ wounds than in Angptl4^+/+^ wounds (Fig. 1A, Table 2), suggesting delayed wound closure in Angptl4^−/−^ mice. Dendritic cells are important to regain homeostasis upon tissue injury [20].

**Table 3:** Distribution of monocytes and monocyte-derived cells in Angptl4^+/+^ and Angptl4^−/−^ wounds.

	1-dpw		5-dpw	
Monocytes/monocyte-derived macrophages	Angptl4^+/+^	Angptl4^−/−^	Angptl4^+/+^	Angptl4^−/−^
Ly6C^hi^CCR2^lo^CX3CR1^lo^	5.41	3.75	1.34	0.92
CCR2^hi^CX3CR1^lo^	2.06	1.96	4.79	2.11
CCR2^lo^CX3CR1^hi^	0.23	0.30	3.76	0.81

The numbers of each of these cell types are expressed as a percentage of the total cells passing QC for that sample, representing CD45^+^ immune cells. Gene expression values are expressed based on the normalized, log-transformed, and scaled count values.

The dynamics between neutrophils and macrophages are important in the transition from inflammation to the proliferative phase of wound healing. Monocyte-derived macrophage numbers peak during the inflammatory phase and before declining during tissue formation [21]. Infiltrated neutrophils undergo apoptosis and are phagocytosed by macrophages. This dynamic is affected by Angptl4 deficiency. Our analyses revealed prolonged neutrophil persistence with markedly reduced mature macrophages in Angptl4^−/−^ mice, which arose from impaired monocyte to macrophage differentiation. It also points to an impaired transition from inflammation to the proliferative phase of wound healing in Angptl4^−/−^ wounds.

### Angptl4 regulates the expression of interferon-activated gene 202b via S6K, FAK, and CDK signaling networks

**Fig. 2:**
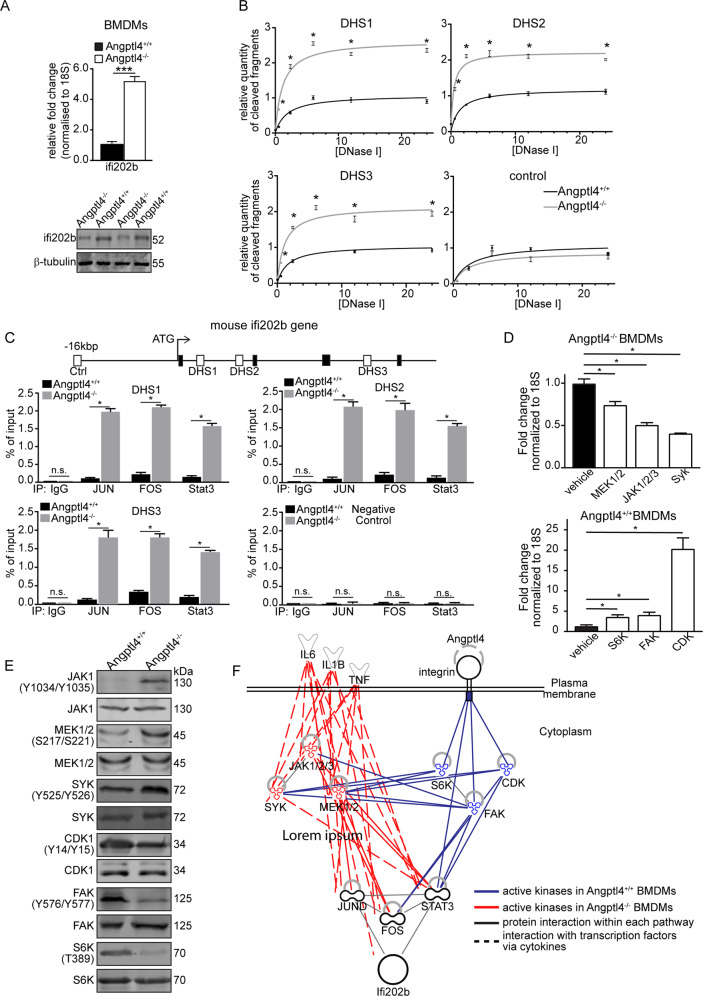
Signaling network regulating mouse ifi202b in monocytes. **A** Relative expression of ifi202b mRNA (top) and protein (bottom) in bone marrow-derived monocytes (BMDMs) from Angptl4^+/+^ and Angptl4^−/−^ mice. **B** Graphs show the quantification of released DNA fragments at the identified DNAse I hypersensitivity site (DHS) using Angptl4^+/+^ and Angptl4^−/−^ BMDMs. A control region that has no known DHS was also quantified as a negative control. **C** Chromatin immunoprecipitation using antibodies against *p*-Jun, *p*-Fos and *p*-Stat3. ChIP was performed using Angptl4^+/+^ and Angptl4^−/−^ BMDMs. **D** Relative fold change of ifi202b mRNA in either Angptl4^+/+^ or Angptl4^−/−^ BMDMs after kinase inhibitor screening. **E** Representative immunoblots of total and phosphorylated kinases in Angptl4^+/+^ and Angptl4^−/−^ BMDMs. Loading controls were from the same samples. **F** Connectivity network of the identified kinases involved in the increased (red) or suppressed (blue) expression of ifi202b in monocytes. Blue and red lines indicate the possible networks by which the kinases exert their activating or repressive effects. For panels **A−D**, data are from *n* = 3 independent experiments with triplicate. *p < 0.05, ***p < 0.001. n.s. not significant.

Our examination of the DEGs revealed that the expression of heme-binding protein 1 (Hebp1) and interferon activated gene 202B (ifi202b) was consistently elevated in 1- and 5-dpw Angptl4^−/−^ compared with Angptl4^+/+^ monocytes and macrophages, but not in other immune cell types (Table S1). While the expression of Hebp1 was higher than ifi202b in monocytes, its expression level was unchanged between monocytes and macrophages. Only the expression of ifi202b was further increased from monocytes to macrophages, overtaking Hebp1 and thus suggesting a potential role in monocyte to macrophage differentiation. We further confirmed the increased mRNA and protein expression of ifi202b in Angptl4^−/−^ BMDMs (Fig. 2A**)**.

Little information is known about the transcriptional regulation of the ifi202b gene. Thus, we first identified the regulatory sites in the mouse Ifi202b gene. Three DNAse I hypersensitive sites (DHS 1−3) are potential regulatory sites in ifi202b, as suggested by data from the ENCODE database [22] (Fig. S6). All DHS 1−3 have increased susceptibility to DNAse I in Angptl4^−/−^ BMDMs compared with Angptl4^+/+^ BMDMs, indicating increased chromatin accessibility (Fig. 2B). In silico analysis of DHS 1−3 identified putative binding sites for Jun, Fos, and Stat3. ChIP analysis confirmed the increased occupancy of these transcription factors at DHS 1−3 in Angptl4^−/−^ BMDMs compared with Angptl4^+/+^ BMDMs (Fig. 2C). Next, we performed an unbiased 133-kinase inhibitor screen using BMDMs to decipher signaling pathways responsible for Angptl4-mediated regulation of ifi202b expression. Kinase inhibitors that decrease the elevated expression of ifi202b in Angptl4^−/−^ BMDMs indicate that their targets are involved in the signaling cascade. Similarly, kinase inhibitors that upregulate ifi202b expression in Angptl4^+/+^ BMDMs indicate that their targets suppress ifi202b expression (Fig. S7). We identified three kinases that significantly down- and upregulated the expression of ifi202b in Angptl4^−/−^ and Angptl4^+/+^ BMDMs, respectively (Fig. 2D). In the presence of Angptl4, we observed high S6K, FAK, and CDK activation that suppressed the expression of ifi202b. We also identified increased phospho-activated Jak1/2/3, Mek1/2, and Syk kinases in Angptl4^−/−^ BMDMs that activate Jun, Fos, and Stat3 transcription factors (Fig. 2E). Integrating these data with protein interaction networks from the STRING database, we revealed the Angptl4-mediated regulatory networks of the ifi202 gene in monocytes (Fig. 2F).

### Ifi202b regulates monocyte differentiation to macrophages

**Fig. 3:**
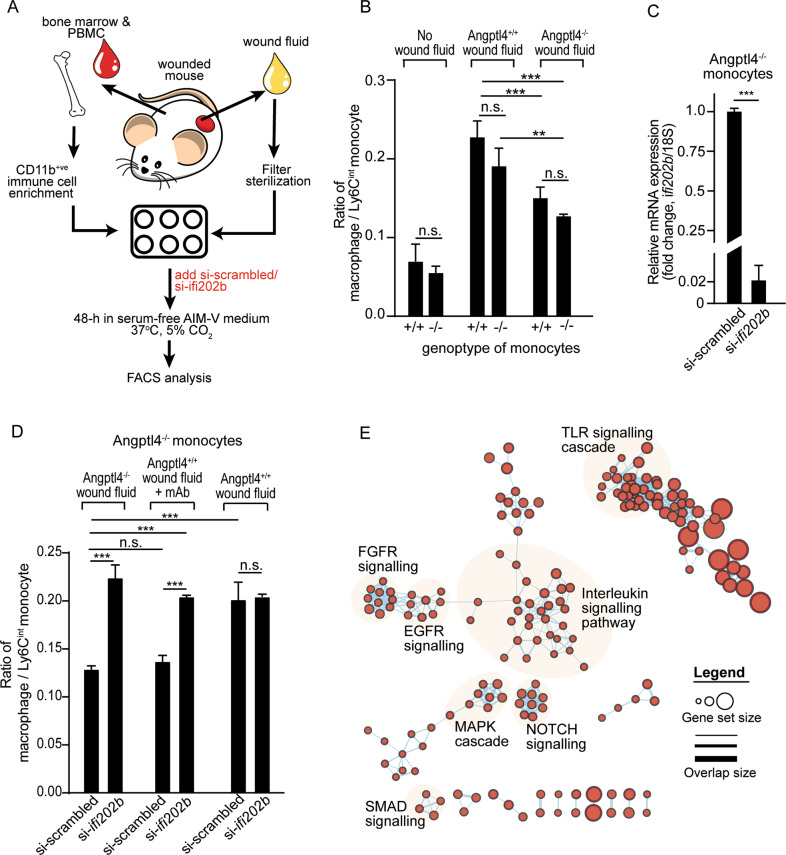
Ifi202b silencing promotes monocyte differentiation to macrophages. **A** Schematic illustration of in vitro monocyte culture supplemented with wound exudate to mimic the in vivo wound microenvironment. PBMC, peripheral blood mononuclear cells. **B** Ratio of macrophage-to-Ly6C^int^ monocytes in Angptl4^+/+^ and Angptl4^−^^/−^ monocytes treated with the indicated wound fluids. Values represent mean ± SD. **C** Relative mRNA expression ifi202b in Angptl4^−/−^ monocytes challenged with Angptl4^−/−^ wound fluid and treated with si-ifi202b. Gene expression was normalized to 18 S rRNA. **D** Ratio of macrophage-to-Ly6C^int^ monocytes in Angptl4^−/−^ monocytes cultured in the indicated wound fluids. Endogenous expression of ifi202b was suppressed by si-ifi202b. si-scrambled served as negative control. mAb denotes blocking anti-cAngptl4 monoclonal antibodies. **E** Functional GSEA of the interaction network associated with ifi202b. Highly enriched cellular functions/processes are highlighted. For panels **B−D**, data are from *n* = 3 independent experiments with triplicate. **p < 0.01, ***p < 0.001. n.s. not significant.

In the wound milieu, cell fate is guided by microenvironmental cues. For wounds, much of this cue is captured in the wound fluid [23]. Therefore, monocytes isolated from the peripheral blood and bone marrow were cultured in wound exudate to mimic in vivo differentiation conditions (Fig. 3A, S8A). Regardless of the mouse genotype, Angptl4^+/+^ wound fluid induced a higher macrophage to Ly6C^int^ monocyte ratio than Angptl4-deficient wound fluid, recapitulated the in vivo Angptl4^+/+^, Angptl4^−/−^, Angptl4^lysM−/−^ wound microenvironments previously shown and confirmed paracrine cell-matrix communication (Fig. 3B, S8B). The silencing of ifi202b in Angptl4^−/−^ monocytes, whose endogenous ifi202b expression was elevated, rescued the impaired monocyte-to-macrophage transition of Angptl4^−/−^ monocytes in wound fluid from Angptl4^−/−^ wounds or Angptl4^+/+^ wound fluid treated with anti-cAngptl4 monoclonal antibody (mAb; clone 3F4F5) (Fig. 3C-D). This mAb has been shown previously to block the action of cAngptl4 [24]. However, in Angptl4^+/+^ wound fluid, the suppression of ifi202b in Angptl4^−/−^ monocytes did not further induce monocyte differentiation compared with Angptl4^−/−^ monocytes with scrambled siRNA (Fig. 3D). These observations indicate that Angptl4 and ifi202b act along the same modulatory pathway. Ifi202 modulates the transcriptional activity of several transcription factors, such as NF-κB and AP-1 [25–28], that likely impact multiple gene networks involved in cell fate and functions. Indeed, GSEA and IPA functional analyses revealed that ifi202b-associated canonical pathways and functions involved in the inflammatory response and monocyte cell fate were enriched, such as interleukin, TLR, SMAD, and NOTCH signaling (Fig. 3E, S8C) [29, 30]. Taken together, our findings clearly highlight that ifi202b, whose expression is regulated by Angptl4, is a key gatekeeper in monocyte to macrophage differentiation during wound healing.

## Discussion

Angptl4 has been implicated in many inflammatory diseases, such as inflammation-induced lung tissue damage [24, 31] and wound healing [9, 16]. The relationship between Angptl4 and inflammation is context-dependent. However, the precise role of Angptl4 in immune cells remains unclear. We showed that Angptl4 in wounds regulates monocyte differentiation to macrophages and coordinates the clearance of neutrophils, thus facilitating the transition from the inflammatory to the proliferative phase of wound healing. There were fewer monocyte-derived macrophages in the absence of Angptl4 in the wound microenvironment, resulting in a prolonged neutrophil presence. The underlying mechanism involves a kinome network that modulates the expression of ifi202b, a transcriptional modulator, to affect multiple pathways involved in cell fate and the inflammatory response.

Macrophage-mediated phagocytosis of apoptotic neutrophils is a key event for transitioning from the inflammatory to the proliferative phase [4, 32]. This dysregulated dynamism between neutrophils and macrophages contributed to the delayed wound healing and stalled inflammation previously observed in Angptl4^−/−^ mice [9, 16]. Angptl4 deficiency in wounds prolongs neutrophil presence and reduces CCR2- and CX3CR1-expressing monocyte-derived macrophages, which are responsible for promoting wound healing [33]. There were fewer mature monocyte-derived macrophages despite similar infiltration of monocytes into Angptl4^−/−^ wounds than Angptl4^+/+^ wounds, as evidenced by flow cytometric and scRNA-sequencing analyses. Our analysis also confirmed a decreased ability of Ly6C^int^ monocytes to acquire an MHC-II^+ve^ phenotype in Angptl4^−/−^ wounds. Consequently, the persistent neutrophil presence and their associated inflammatory response were detrimental to wound repair, which is a major driver of chronic wounds [34].

Angptl4-deficient wound monocytes and macrophages have elevated expression of ifi202b, which is not observed in other wound immune cells. We revealed that an Angptl4-associated kinome network involving FAK and Syk, among other kinases, modulates the expression of ifi202b. The activation of FAK by Angptl4 was consistent with previous studies showing that Angptl4 interacts with specific integrins to activate integrin-FAK signaling, affecting the cellular response in a context-dependent manner [9, 35]. Syk is a nonreceptor tyrosine kinase highly expressed in hematopoietic cells [36, 37]. The activation of Syk leads to downstream activation of signaling mediators such as phosphoinositide 3-kinase and Rac/Rho signaling that contribute to immune cell responses [243]. An in vitro reconstituted wound microenvironment study showed that wound fluid derived from Angptl4^+/+^ wounds could stimulate monocyte to macrophage differentiation, regardless of monocyte genotype. By silencing ifi202b expression, Angptl4^−/−^ monocytes could overcome impaired differentiation due to Angptl4 deficiency or depletion in wound fluids, resulting in more monocyte-derived macrophages. It is also interesting that Angptl4 deficiency in macrophages increased the secretion of more TNF-α than wild-type macrophages following LPS stimulation [12]. This observation is consistent with a stalled or enhanced inflammation phase in Angptl4-deficient wounds. Our GSEA of ifi202b-associated pathways revealed that signaling mediators, such as MAP kinase, SMAD, and NOTCH signaling mediators, were enriched, which are also crucial regulators of monocyte to macrophage differentiation [30, 38]. Interestingly, the cell fates of Ly6C monocytes and the resultant inflammation are coordinately regulated by TLR and Notch signaling, both of which are linked to ifi202b [29]. Conceivably, the dynamics between the activating and suppressive signaling networks due to infiltrated monocytes interrogating the wound microenvironment fine-tune the expression of ifi202b and, consequently, the ratio between monocytes and monocyte-derived macrophages in the wounds.

Our current study identified a role for Angptl4 in the acute inflammatory response to injury. It is conceivable that Angptl4 also plays a distinct role in adaptive immunity involving T and B cells, which were underrepresented in our skin wounding model. Other chronic inflammatory models, such as atherosclerosis or arthritis, may be more suitable. Of particular interest is systemic lupus erythematosus (SLE). SLE is an autoimmune disease with common clinical manifestations in skin lesions, hyperkeratosis, and scarring [39]. Treatment strategies against SLE generally involve immunosuppression that poorly addresses disease manifestations. It is widely established that genetic predisposition has a key role in susceptibility to SLE [40]. To date, ifi202b is best studied for its role as an SLE susceptibility gene. Interestingly, Angptl4 has been identified as a candidate biomarker for lupus nephritis in patients with SLE [41, 42]. In patient sera, Angptl4 expression was significantly lower than that in healthy controls. Clearly, further investigation will be necessary to confirm a role for Angptl4 in SLE.

In conclusion, Angptl4 in the wound milieu regulates the expression of ifi202b, which acts as a gatekeeper facilitating wound monocyte-to-macrophage differentiation during wound healing.

## Materials and Methods

### Mouse excisional skin wounding

Eight-weeks old wild-type C57BL/6 J male mice (Invivos, Singapore) and Angptl4^−^^/−^ male mice (Genentech UNQ171, Mutant Mouse Regional Resource Center (MMRRC)) were housed in a specific-pathogen-free environment and maintained at 24 °C and 50% humidity with a 12 h light/dark cycle. Under anesthesia, the dorsal skin was depilated and sterilized, and 3 mm excisional wounds were made with a biopsy punch [43]. Power analysis was used to determined sample size. All experiments were performed following the guidelines of the Institutional Animal Care and Use Committee (NTU-IACUC: A0324, A19034).

### In vitro reconstituted wound microenvironment

CD11b^+^ immune cells isolated from peripheral blood mononuclear cells and bone marrow were cultured in serum-free AIM-V^TM^ medium (Gibco) with 2% penicillin/streptomycin at a density of 2 × 10^6^ cells/mL and challenged with 20% (v/v) wound fluids from Angptl4^+/+^ and Angptl4^−^^/−^ mice to mimic the wound microenvironment. Angptl4 neutralizing mAb (10 μg/mL; clone 3F4F5) was added to deplete cAngptl4 in Angptl4^+/+^ wound fluid. After 48 h, cells were retrieved and subjected to FACS analysis using antibodies, as shown in Table S2, based on the gating strategy in Fig. S1A. The macrophage/Ly6C^int^ monocyte ratio was calculated based on (Differentiating macrophages [Population 3] + Macrophages [Population 4])/ Ly6C^int^ monocytes [Population 2].

### Flow cytometry

Wound tissues were dissociated using 1 mg/mL Type 3 collagenase and 10 U/mL DNAsel for 1 h in a 37 °C water bath. Cells were separated through a 30 μm strainer, blocked with 3% BSA containing Fc blocker, and stained with selected antibodies (Table S2). FACS data were collected using BD LSRFortessa X-20 and analyzed on FlowJo.

### Single-cell RNA sequencing and bioinformatic analysis

Cell suspensions from wound tissues were stained with CD45-APC and propidium iodide before sorting on a BD FACSAria^TM^ Fusion. Live immune cells (CD45^+^ PI^-^) were collected and washed with PBS before proceeding to single-cell library preparation. Single-cell droplets and cDNA libraries were prepared using a Chromium Single Cell 3’ V3 kit (10X Genomics) according to the manufacturer’s instructions. The cDNA libraries were sequenced on an Illumina NovaSeq6000. Raw sequencing reads were aligned to the mm10 mouse reference genome using Cell Ranger (v3.1.0) to generate single-cell count matrices that were normalized, integrated, and annotated using Seurat (v3.0) [44].

### Statistical analysis

Statistical analysis was performed using Prism 9 (GraphPad Software Inc). Unless otherwise stated, statistical significance was calculated using unpaired Student’s *t-*test for comparisons between two groups or one-way ANOVA followed by Tukey’s test for comparisons between multiple (>2) groups. A *p-*value of <0.05 was considered statistically significant. Power analysis was used to determined sample size.

## Supplementary information

Supporting Information and Data

RAW Westerns

Reproducibility checklist

## Data Availability

Single-cell RNA-seq data were deposited at the Gene Expression Omnibus (GEO; Accession number: GSE186986). More detailed methods are presented in the Supporting Information.
