# Supplementary material for: Single-cell analysis of skin immune cells reveals an Angptl4-ifi20b axis that regulates monocyte differentiation during wound healing
Source: Cell Death Dis. 2022 Feb 24;13(2):180. doi: 10.1038/s41419-022-04638-7 (PMC8873364; doi:10.1038/s41419-022-04638-7)
Supplement: Supplementary file 1 — Supporting Information and Data [file 41419_2022_4638_MOESM1_ESM.pdf]

Supporting Information

Single-cell analysis of skin immune cells reveals an Angptl4-ifi20b axis that regulates monocyte differentiation during wound healing.

Wei Kiat Jonathan WEE[1,2], Zun Siong LOW[1], Chin Kiat OOI[2], Benjamin Patrana HENATEGALA[2], Zhi Guang Ridley LIM[2], Yun Sheng YIP[1], Marcus Ivan Gerard VOS[1], William Wei Ren TAN[1], Hong Sheng CHENG[1], Nguan Soon TAN[1,2*]

[1]Lee Kong Chian School of Medicine, Nanyang Technological University Singapore, 11 Mandalay Road, Singapore 308232.

[2]School of Biological Sciences, Nanyang Technological University Singapore, 60 Nanyang Drive, Singapore 637551.

Supplemental Figure legends

Figure S1. Gating strategy and FACS analysis of skin immune cells from Angptl4$^{+/+}$ and Angptl4$^{-/-}$ wounds.

(A) Representative plots outlining the gating strategy to identify neutrophils and the subpopulations of monocytes and macrophages. Cells were first gated for forward and side scatter and singlets. Neutrophils were identified by CD11b^{hi} myeloid cells with Ly6G^{+ve}MHC-II^{-ve}. The Ly6G^{-ve}CD11b^{hi} myeloid subpopulation was further sorted based on the expression of Ly6C and MHC-II. Four major subpopulations of monocytes and monocyte-derived macrophages were identified in our excisional skin wounds.

(B) Representative flow cytometry plots showing the temporal changes in monocytes and monocyte-derived macrophages in Angptl4$^{+/+}$ and Angptl4$^{-/-}$ excisional wounds at the indicated days post-wounding (dpw). Data are from n = 3 independent experiments with triplicate

Figure S2. Angptl4 deficiency does not affect monocyte recruitment and infiltration.

(A) Cytokine profiles of wound exudates from Angptl4$^{+/+}$ and Angptl4$^{-/-}$ wounds at 8 h and 1 day post-wounding.

(B) Graph showing the fold change in the fluorescent signal of migrated PBMCs from Angptl4$^{+/+}$ and Angptl4$^{-/-}$ mice. Monocyte chemotaxis assay was performed using 25 nM MCP-1 on the migration of prelabeled cells.

(C) Angptl4$^{LysM-/-}$ conditional knockout mice had similar percentages of neutrophils and mature macrophages compared with Angptl4$^{f/f}$ wounds. Graphical representation of Ly6G^{hi} neutrophils and Ly6C^{lo}MHC-II^{hi} macrophages in wounds at 5 dpw.

Data are from n = 3 independent experiments with 3 mice at each time point. *$p < 0.05$, n.s. denotes not significant.

Figure S3. Gating strategy for the isolation of viable CD45^{+} immune cells for single-cell RNA-seq.

(A) Single-cell suspensions were obtained from Angptl4$^{+/+}$ and Angptl4$^{-/-}$ excisional wounds using optimized tissue dissociation protocols. 1-dpw wounds were pooled from three mice, and 5-dpw wounds were pooled

from five mice. Cells were first gated using forward and side scatter to exclude debris and cell doublets, viable cells were gated as propidium iodide-negative cells, and immune cells were gated as CD45-positive cells.

(B) Quality control metrics of the collected viable cells. Single-cell libraries were prepared using 5000-6000 cells as input for each sample. After sequencing, alignment, and filtering, we obtained combined data from a total of 19579 cells.

Figure S4. The immune cell landscape in mouse excisional skin wounds.

(A) Unified UMAP dimensionality reduction plot showing aggregated $CD45^{+}$ immune cells from $Angptl4^{+/+}$ and $Angptl4^{-/-}$ wounds at 1 and 5 dpw. Cells across genotypes and timepoints were aligned using a mutual nearest neighbor approach.

(B) An in-depth characterization of immune cell lineages with the ImmGen database. The mean normalized gene expression value of each input gene is expressed as a heatmap. Each row represents an input gene, and each column represents an ImmGen microarray dataset grouped by cell type (colored).

Figure S5. Neutrophils during wound healing consist of two subpopulations.

(A) Heatmap showing the differential gene expression between the two neutrophil clusters 0 and 1. Differentially expressed genes were selected based on a 1.5-fold change cut off. The UMAP plot of neutrophils from aggregated samples showed the enrichment of protumorigenic and proinflammatory factors in neutrophil clusters 0 and 1, respectively.

(B) The expression of canonical genes known to delineate the differentiation of monocytes to macrophages: Ly6c, MHC-II genes (H2-Aa and Cd74), and Adgre1 (F4/80)

(C) Expression of known markers of resident tissue macrophages in the skin: Langerin (Cd207) and Siglec-1 (Cd169) are shown.

Figure S6. Identification of novel regulatory regions in ifi202b using ENCODE.

Publicly deposited mouse DNAse-seq data available on ENCODE across all tissues and cell lines were studied. The datasets containing DNAse I hypersensitive sites on ifi202b are graphically shown here, using a genomic visualization tool by the UCSC Genomics Institute. The chromosomal location and intron-exon structure of ifi202b are displayed to scale. Each row shows the normalized fold change signal from DNAse-seq data. Dataset identifiers for the displayed ENCODE datasets are listed next to each row.

Figure S7. A kinase inhibitor screen for mediators involved in ifi202b regulation in BMDMs.

(A) A schematic overview of the kinase inhibitor screen. The expression of ifi202b was low in Angptl4$^{+/+}$ BMDMs and was strongly upregulated in Angptl4$^{-/-}$ BMDMs (left column). Kinase inhibitors that upregulate ifi202b expression in Angptl4$^{+/+}$ BMDMs are involved in the Angptl4-mediated suppression of ifi202b (middle column). Kinase inhibitors that reduce the increased expression of ifi202b in Angptl4$^{-/-}$ BMDMs are involved in the upregulation of ifi202b as a result of Angptl4 deficiency.

Figure S8. *In vitro* monocyte culture with wound fluid to mimic the wound microenvironment

(A) Positive selection of CD11b^{+ve} cells from murine peripheral blood and bone marrow.

(B) Representative flow cytometry plots showing monocytes and monocyte-derived macrophages in the indicated wound fluid. **G**ating strategy follows that in Figure S1A. The four populations were as follows: 1= Ly6C^{hi} monocytes; 2=Ly6C^{int} monocytes; 3=differentiating macrophages; and 4=macrophages.

(C) IPA analysis reveals enriched gene ontologies in inflammatory response, growth factor signaling, cell–cell and cell-matrix interactions and cell signaling.

Supplemental Materials

Table S1: Differentially expressed genes between Angptl4$^{+/+}$ and Angptl4$^{-/-}$ monocytes and macrophages from wounds.

1-dpw				5-dpw			
monocytes		macrophages		monocytes		macrophages	
Gene	FC	Gene	FC	Gene	FC	Gene	FC
Hebp1	3.51	**Ifi202b**	3.26	Emp1	3.53	**Ifi202b**	2.23
Emp1	3.29	Hebp1	2.72	Hebp1	2.14	Hebp1	2.02
Gm10036	1.92	Isg15	1.86	Uba52	1.83	Cxcl3	1.90
**Ifi202b**	1.76	Marcksl1	1.70	**Ifi202b**	1.65	Isg15	1.89
Sh3bgrl3	0.59	Arhgdib	0.67	Gm10076	1.50	Mmp9	1.68
Eps8	0.48	Trem2	0.65	Eps8	0.57	Uba52	1.65
		Hmox1	0.64			Rsad	1.57
		Pdpn	0.61			Cxcl2	1.54
		Eps8	0.57			Pltp	0.66
		Tnfrsf9	0.51			Sgk1	0.66
						Pgk1	0.65
						Selenbp1	0.65
						Smpdl3a	0.64
						Serpine1	0.63
						Gpi1	0.60
						Fl1r	0.60
						Trem2	0.59
						Mt2	0.43
						Mt1	0.41
						Ltc4s	0.37

Differential gene expression between Angptl4$^{+/+}$ and Angptl4$^{-/-}$ monocytes and macrophages was conducted using the nonparametric Wilcoxon rank sum test. All statistically significant differentially expressed genes are shown in this table. FC denotes the fold change in Angptl4$^{-/-}$ mice compared with Angptl4$^{+/+}$ mice.

Table S2: Antibodies used for FACS analysis.

Name	Cell type	Company	Catalog Number
Ly6G-FITC	Neutrophil	Miltenyi	REA526
MHC-II-PE	Macrophage	Miltenyi	REA528
CD11b-APC	Myeloid cell	Miltenyi	M1
CD45-APC	Leukocyte	Miltenyi	REA737
Ly6c-BV421	Mouse monocyte	Biolegend	128032

Supplemental Methods

Bioinformatic analysis of single-cell RNA sequencing data

The single-cell RNA sequencing data were analyzed using the R package Seurat (v3.0) (1). Datasets were first screened for quality control by excluding cells with fewer than 200 and more than 4500 detected genes to remove empty droplets and doublets. Cells with more than 5% mitochondrial genes were excluded from subsequent analysis to remove cells of poor viability. Genes detected in less than 3 cells were also excluded from further analysis. Gene expression data were then normalized using a regularized negative binomial regression method, and a subset of features that exhibited high cell–cell variation was calculated using a variance stabilizing transformation with SCTransform. These variable features were used for subsequent analyses.

The four datasets (WT-1Day, WT-5Day, KO-1Day, and KO-5Day) were integrated into a unified dataset. Briefly, a shared gene correlation structure conserved between all datasets was learned using canonical correlation analysis (CCA). These datasets are then aligned into a conserved low-dimensional space using dynamic time warping algorithms to normalize for differences in feature scale. Finally, the integrated data were used for further downstream analysis and visualization.

Clustering is performed using a K-nearest neighbor (KNN) graph approach, followed by modularity optimization using the Louvain algorithm. Nonlinear dimensional reduction using UMAP was used to visualize the data. Cell type annotation was performed using the ImmGen database from celldex (v1.2.0) (2).

Differential gene expression testing was performed using the nonparametric Wilcoxon rank-sum test. For the identification of cluster biomarkers, differential expression testing was conducted between each cluster compared against all remaining clusters.

Pseudotime trajectory analysis

Pseudotime trajectory analysis was performed using Monocle3 (3). Preprocessed 10X genomics count barcode matrices were supplied as input to Monocle. Monocyte and macrophage populations were identified from the UMAP plots and subsets into a separate data file. A single-cell trajectory was then

learned using Monocle's algorithm, with the monocyte cell cluster specified as the root node (pseudotime 0) of the trajectory. Cells were then ordered according to their calculated pseudotime.

RNA Extraction and Real-time Quantitative PCR

Total RNA was first extracted using TRIzol (Invitrogen, USA) reagent according to the manufacturer's instructions. After phase separation and precipitation, RNA was transferred to an RNA column-based kit (Research Instruments). Total RNA (1 µg) was quantified using a NanoDrop ND-1000 (Thermo Scientific) and reverse transcribed to cDNA using qScriptTM Reverse Transcription Supermix (Quanta Bio) according to the manufacturer's protocol. qPCR was performed using a Bio–Rad C1000 Thermal Cycler equipped with a CFX96 Real-Time System. Reactions were prepared using PerfeCTa SYBR Green Mix (Quanta Bio) according to the manufacturer's instructions. The primers used are outlined in **Table S**.

Table S3: **List of primers used for qPCR experiments.**

Experiment	Name	Forward Primer	Reverse Primer
DHS, ChIP	DHS1	AAG GCA CTT CAT GCT TTT TAG CC	TTC TGC CAC TGT GAA AGA GAC CA
DHS, ChIP	DHS2	GAA CAT GGA TTT GCA GGT GTG AG	AGA TCC CAA ACC TCA GCT TGC AT
DHS,ChIP	DHS3	AGA CCC AGT GGC ATA CTT TCT CA	CCC AAT TTC CAG TTT TCT AAC AGG
DHS, ChIP	DHS Control	CTA GGC CAC AGA ATT GAA AAG ATC	GTA GGT GGA AAT TCT AGC ATC AT
ChIP	Negative Control	GTT ATA ATA CAC CTC ACC ATA TCT GGG	CGA TCA TCT CTG CTT CAT CTC C
qPCR	Ifi202b	GAC CCC TTC CAG TGA TTC ATC T	ACA GCA CCT TTG CTA ATG TTC T
qPCR	18S rRNA	GTA ACC CGT TGA ACC CCA TT	CCA TCC AAT CGG TAG TAG GG
qPCR	Angptl4	TCC AAC GCC ACC CAC TTA C	TGA AGT CAT CTC ACA GTT GAC CA

Wound fluid collection

Six-millimeter excisional wounds were created on the dorsal skin of Angptl4$^{+/+}$ or Angptl4$^{-/-}$ mice and covered with stacks of round absorbent papers. The wounds were then covered with parafilm and secured with Tegaderm^{TM} Film Dressing. The absorbent papers were collected and replaced every 10-12 hours for up to two days. The volume of wound fluid absorbed by the papers was estimated based on weight change. Absorbent papers from the same genotype were pooled, and an equal volume of serum-free AIM-V medium with 2% penicillin/streptomycin was added. The mixture was sonicated for 15 minutes

in an ice-cold water bath. Wound fluid was collected from the absorbent papers and sterile filtered. The protein concentration was determined with the Bradford assay.

Isolation of bone marrow and PBMC-derived monocytes

Immune cells were purified from PBMCs and bone marrow using Percoll at a 1.07 g/mL density. Positive selection of $CD11b^+$ cells was performed with CD11b MicroBeads (Miltenyi Biotec) according to the manufacturer's protocol. The CD11b-enriched fraction was used for *in vitro* monocyte culture.

RNA interference

Angptl4 silencing was performed using ON-TARGETplus SMARTpool siRNA (Horizon Discovery) targeting mouse Angptl4. RAW264.7 macrophage cells were transfected with siRNA using DharmaFECT 1 reagent (Horizon Discovery) according to the manufacturer's instructions. Transfection efficiency using DharmaFECT 1 reagent was optimized using siGLO reagent to achieve more than 80% transfection efficiency. Knockdown of *ifi202b* in bone marrow and PBMC-derived monocytes was achieved with Accell mouse *ifi202b* SMARTpool siRNA (Horizon Discovery) at a final concentration of 1 µM. Control cells were transfected with si-scrambled. Knockdown efficiency was determined by qPCR.

Western blot

Cells were lysed in ice-cold RIPA buffer and centrifuged at 12,000 g for 10 minutes. The supernatant was collected, and Laemmli loading buffer was added. Proteins were run on a 10% SDS–PAGE gel and transferred to a PVDF membrane. The membrane was blocked for 1 hour with Li-Cor Odyssey blocking buffer and incubated overnight with the respective primary antibodies at 4°C. The membrane was washed 3 times with TBST before incubation with secondary antibody for 1 hour at room temperature. The membrane was washed 3 times with TBST, dried, and scanned using an Odyssey CLx scanner (Licor). Antibodies against ifi202b and β-tubulin were purchased from Santa Cruz and Developmental Studies Hybridoma Bank, respectively. Anti-mouse Angptl4 antibody was produced in-house as reported (4, 5).

Cytokine assay

For the *in vivo* wound fluid cytokine assay, excisional wounds were performed on mice as described above. Sterile blotting sponges were placed at the wound incision site, and the wound area was protected with an occlusive Tegaderm dressing (3 M). Sponges were collected at the indicated time points, and wound fluid was extracted through centrifugation at 10,000 g for 5 minutes. The collected wound fluid was diluted 2-fold and analyzed using the MD31 31 31-plex mouse cytokine array (Eve Technologies).

Kinase Inhibitor Screening

Angptl4$^{-/-}$ BMDMs were seeded on a 6-well plate prior to the experiment. Cells were treated with a panel of 95 kinase inhibitors from the SYN2103 kinase inhibitor library (SYNKinase) with 1-well of vehicle control. Cells were incubated with the inhibitors for 4 hours before total RNA was extracted, and the mRNA expression of *ifi202b* was quantified using qPCR. The kinase inhibitor library consisted of the following inhibitors, and cells were treated at IC50 concentrations (6) (**Table S4**).

Table S4: List of kinase inhibitors and the concentration used in nM.

	1	2	3	4	5	6	7	8	9	10	11	12
A	DMSO	R428 (14)	Purvalan ol B (9)	NVP-BGJ398 (10)	Tofacitini b (91)	TAK-715 (30)	PP242 (8)	GDC-0941 (42)	(R)-BI-2536 (5)	GNF-5837 (42)	Axitinib (2)	MK-2461 (2.5)
B	A-674563 HCl (16)	ABT-737(40)	BMS387 032 HCl(62)	PD-0173074 (25)	CC-401 HCl(50)	AS7030 26(11)	Rapamy cin (2)	IC87114 (500)	PF43139 6 (31)	GW4417 56 (2)	Bosutini b (1.2)	Motesani b (6)
C	MK-2206 2HCl (65)	_Imatinib mesylate (600)	PF04777 36 (0.49)	Quizartin ib (4.2)	Masatini b (540)	CI-1040 (17)	XL388 (10)	PIK-75 (6)	PLX403 2 (31)	GDC-046 (13.3)	Cabozan tinib (14.3)	Ponatini b (6)
D	LDK378 (0.2)	GCI1746 (2)	CX-4945 (1)	SB-216763 (35)	AMG-47a (10)	Selumeti nib (50)	BX795 (15)	PIK-90 (60)	GSK429 286A (190)	Tivozani b (1)	Dasatini b (1)	Regoraf enib (46)
E	S-99 (1)	PD1739 55-Analog1 (10)	Gefitinib (30)	Mubritini b (6)	BMS-5 (8)	Crizotini b (24)	GSK260 6414 (3.2)	TGX221 (50)	BI-D1870 (31)	JNJ-3815847 1 (50)	CHIR-258 (36)	Sorafeni b (90)
F	CYC116 (70)	Alvocidib (40)	Lapatinib (11)	LY27845 44 (3)	RWJ-67657 (200)	PF04217 903 (5)	AS-252424(33)	YM2016 36 HCl (33)	PF-0470867 1 (160)	SAR131 675 (235)	E7080 (Lenvati nib) (5.2)	SU-6668 (Orantini b) (8)
G	SNS-314 (31)	AT-7519 HCl (210)	PF-562271 (1.5)	Momeloti nib (20)	SB2021 90 (100)	Foretinib (1)	BKM120 (2)	SMI-4a (17)	R406 (41)	Pazopan ib (146)	Intedani b (34)	Sunitinib malate (80)
H	Tozasert ib (70)	Palbocicl ib Isethiona te (16)	Brivanib (25)	TG1013 48 (3)	SD169 (4)	MPI0479 605 HCl (35)	GDC-0032 (10)	Bisindoyl maleimid e X HCl (15)	AMG-Tie2-1 (1)	Amuvati nib (81)	Linifanib (66)	H12_Ta ndutinib (100)

Chromatin Immunoprecipitation

Angptl4$^{+/+}$ or Angptl4$^{-/-}$ mature BMDMs were seeded on a 10 cm cell culture dish. Chromatin immunoprecipitation was performed as previously described (7) (**Table S5**). Purification of immunoprecipitated DNA was performed using the E.Z.N. A gel extraction kit (Omega Biotek) was used according to the manufacturer's instructions. Transcription factor binding was quantified using qPCR with primers against sites of interest and expressed using the percent input calculation (8), as follows: *Percent input = 100*2^(Adjusted input - Ct (IP), where adjusted input is Ct(Input)-6.644 (1%)*

Table S5: List of antibodies used for chromatin immunoprecipitation experiments.

Name	Company	Catalog Number
pJun	Cell Signalling Technology	9261
pFos	Cell Signalling Technology	5348
pStat3	Cell Signalling Technology	9145

DNAse I Hypersensitivity Assay

Cells were detached with a 0.25% trypsin solution, pelleted, and resuspended in DNAse lysis buffer (10 mM Tris-HCl pH 7.4, 0.1% Triton X-100, 10 mM NaCl, and 3 mM $MgCl_2$). The samples were treated with either 0 U, 0.6 U, 2.4 U, 6 U, 12 U, or 24 U of DNAse I in a 37°C water bath for 5 minutes to digest the chromatin. Two volumes of stop buffer (10 mM Tris-HCl pH 7.4, 10 mM NaCl, 10 mM EDTA, and 0.15% SDS) were added to halt the reaction. DNAse I hypersensitivity assay was performed (9). Digested chromatin was purified using the E.Z.N. A gel extraction kit (Omega Biotek). The digestion of DNase hypersensitive sites was quantified using qPCR primers against DNAse I hypersensitive sites in ifi202b. The relative quantity of digested fragments was plotted against the concentration of DNAse I used.

References

1. Y. Hao *et al.*, Integrated analysis of multimodal single-cell data. *Cell* **184**, 3573-3587 e3529 (2021).
2. D. Aran *et al.*, Reference-based analysis of lung single-cell sequencing reveals a transitional profibrotic macrophage. *Nat Immunol* **20**, 163-172 (2019).
3. C. Trapnell *et al.*, The dynamics and regulators of cell fate decisions are revealed by pseudotemporal ordering of single cells. *Nat Biotechnol* **32**, 381-386 (2014).
4. Y. Y. Goh *et al.*, Angiopoietin-like 4 interacts with matrix proteins to modulate wound healing. *J Biol Chem* **285**, 32999-33009 (2010).
5. P. Zhu *et al.*, Angiopoietin-like 4 protein elevates the prosurvival intracellular O2(-):H2O2 ratio and confers anoikis resistance to tumors. *Cancer Cell* **19**, 401-415 (2011).
6. H. S. Cheng *et al.*, Kinomic profile in patient-derived glioma cells during hypoxia reveals c-MET-PI3K dependency for adaptation. *Theranostics* **11**, 5127-5142 (2021).
7. M. M. K. Lim *et al.*, Targeting metabolic flexibility via angiopoietin-like 4 protein sensitizes metastatic cancer cells to chemotherapy drugs. *Mol Cancer* **17**, 152 (2018).
8. X. Lin, L. Tirichine, C. Bowler, Protocol: Chromatin immunoprecipitation (ChIP) methodology to investigate histone modifications in two model diatom species. *Plant Methods* **8**, 48 (2012).
9. J. Cooper, Y. Ding, J. Song, K. Zhao, Genome-wide mapping of DNase I hypersensitive sites in rare cell populations using single-cell DNase sequencing. *Nat Protoc* **12**, 2342-2354 (2017).

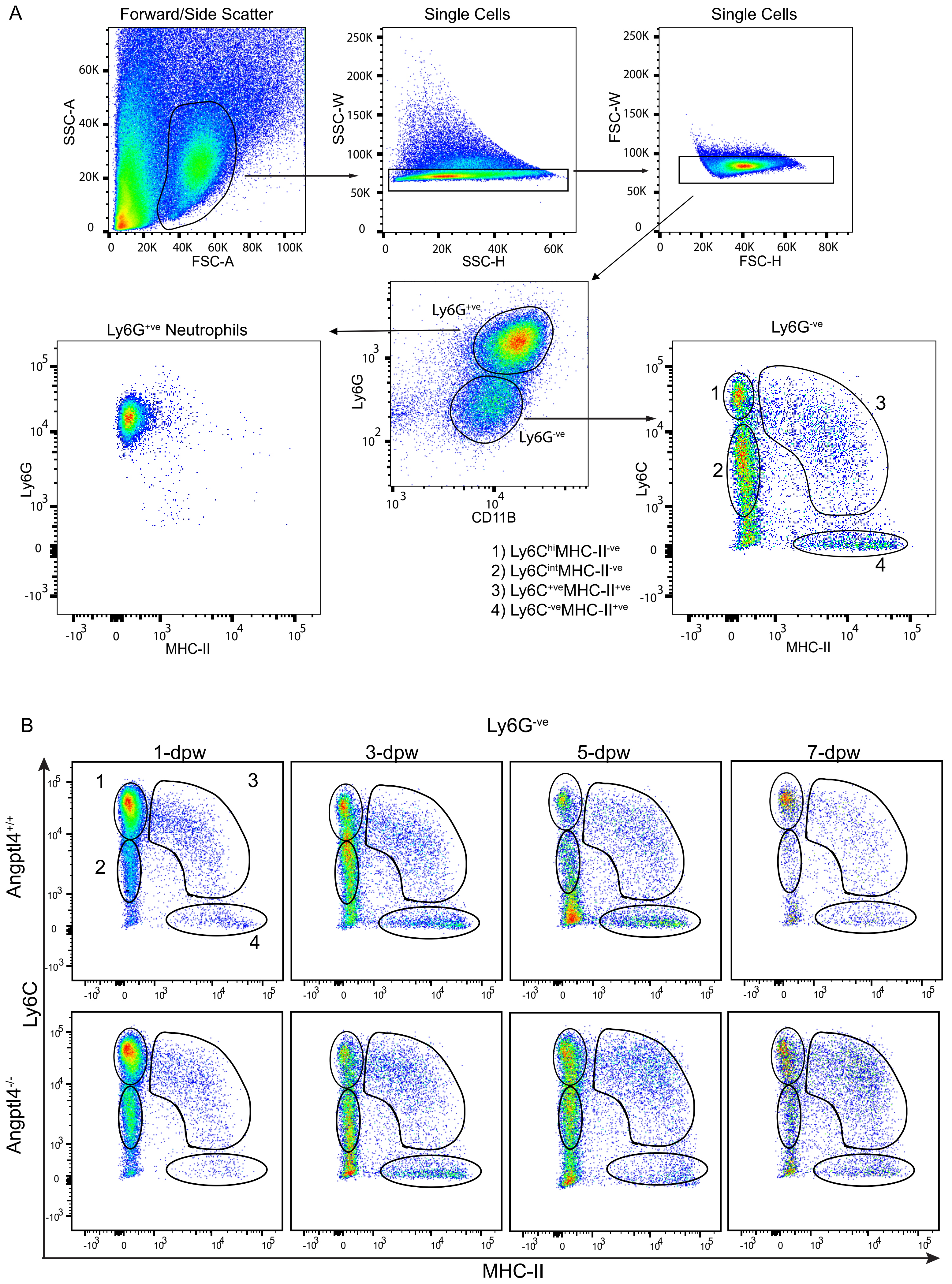
A
Forward/Side Scatter
SSC-A
FSC-A
Single Cells
SSC-W
SSC-H
Single Cells
FSC-W
FSC-H
Ly6G+ve
Ly6G-ve
Ly6G
CD11B
Ly6G+ve Neutrophils
Ly6G
MHC-II
Ly6G-ve
Ly6C
MHC-II
1) Ly6ChiMHC-II-ve
2) Ly6CintMHC-II-ve
3) Ly6C+veMHC-II+ve
4) Ly6C-veMHC-II+ve
B
Ly6G-ve
1-dpw
3-dpw
5-dpw
7-dpw
Angptl4+/+
Angptl4-/-
Ly6C
MHC-II

Wee et al., Figure S2

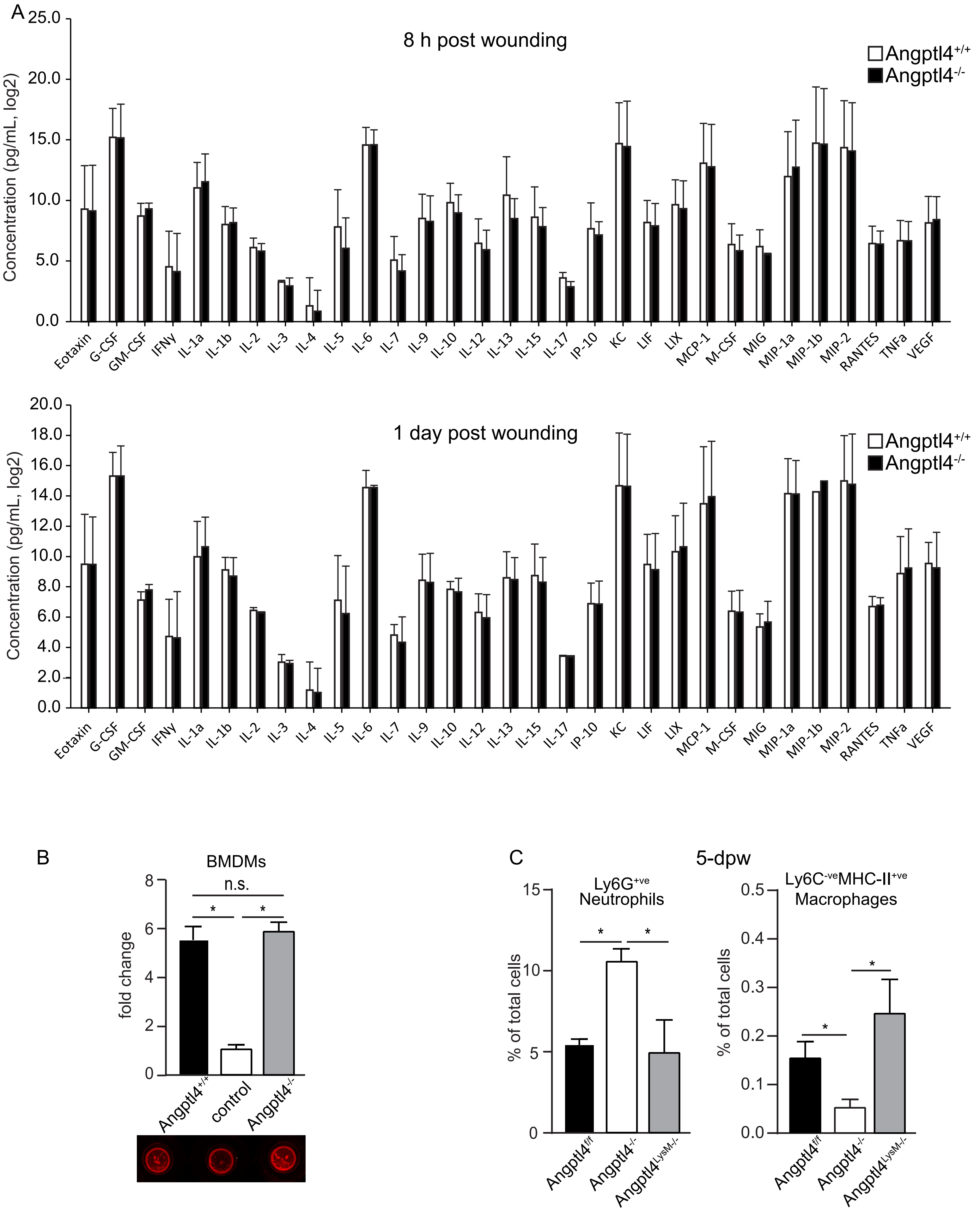

A

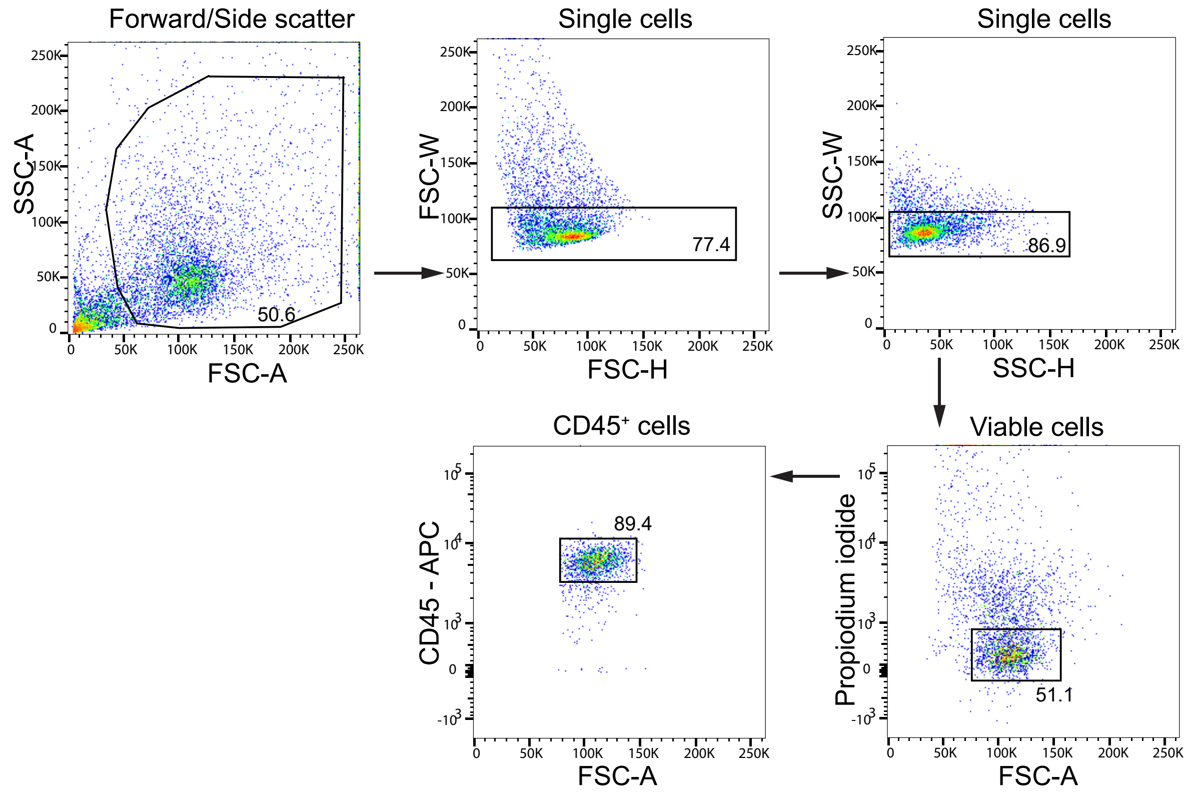

B

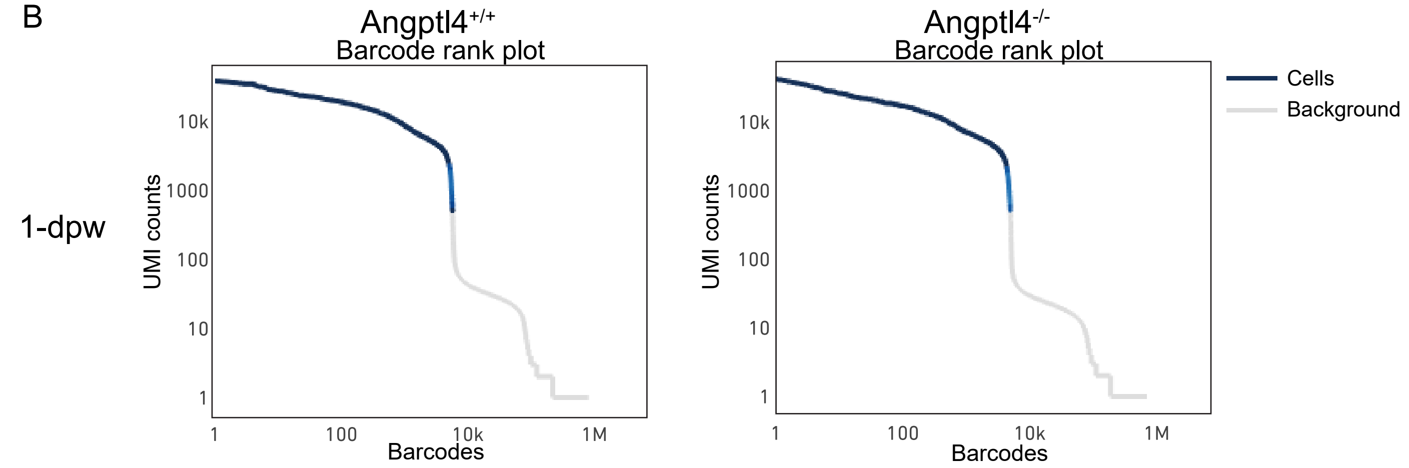

Estimated Number of Cells	5,409
Fraction Reads in Cells	93.80%
Mean Reads per Cell	42,849
Median Genes per Cell	1,185
Total Genes Detected	17,018
Median UMI Counts per Cell	4,953

Estimated Number of Cells	4,805
Fraction Reads in Cells	93.90%
Mean Reads per Cell	36,765
Median Genes per Cell	1,114
Total Genes Detected	16,238
Median UMI Counts per Cell	4,812

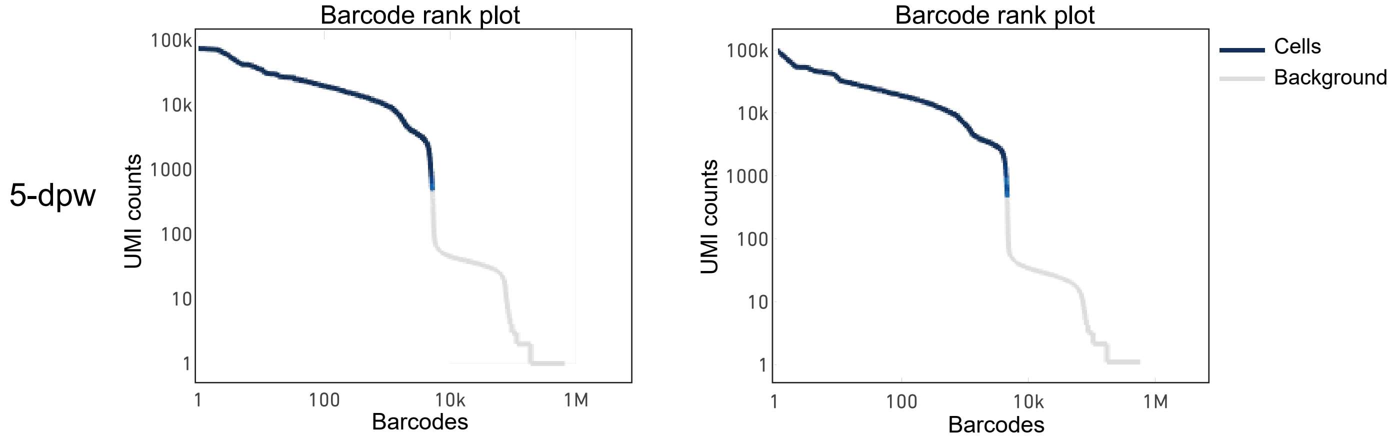

Estimated Number of Cells	5,051
Fraction Reads in Cells	92.60%
Mean Reads per Cell	41,686
Median Genes per Cell	1,276
Total Genes Detected	17,757
Median UMI Counts per Cell	3,829

Estimated Number of Cells	4,770
Fraction Reads in Cells	93.60%
Mean Reads per Cell	54,861
Median Genes per Cell	1,164
Total Genes Detected	17,603
Median UMI Counts per Cell	3,769

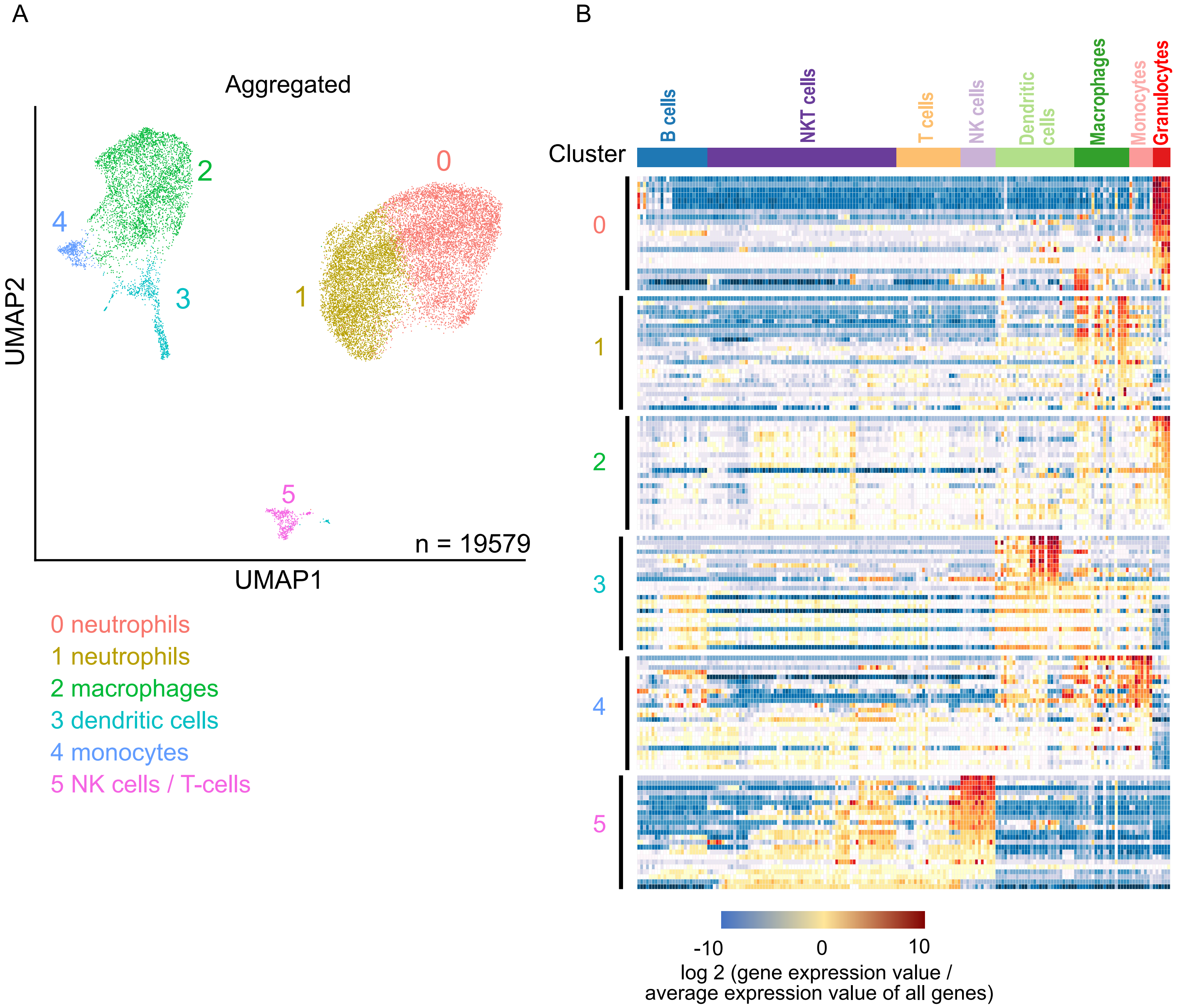
A
Aggregated
UMAP2
UMAP1
n = 19579
0
1
2
3
4
5
0 neutrophils
1 neutrophils
2 macrophages
3 dendritic cells
4 monocytes
5 NK cells / T-cells
B
Cluster
B cells
NKT cells
T cells
NK cells
Dendritic cells
Macrophages
Monocytes
Granulocytes
-10
0
10
log 2 (gene expression value / average expression value of all genes)

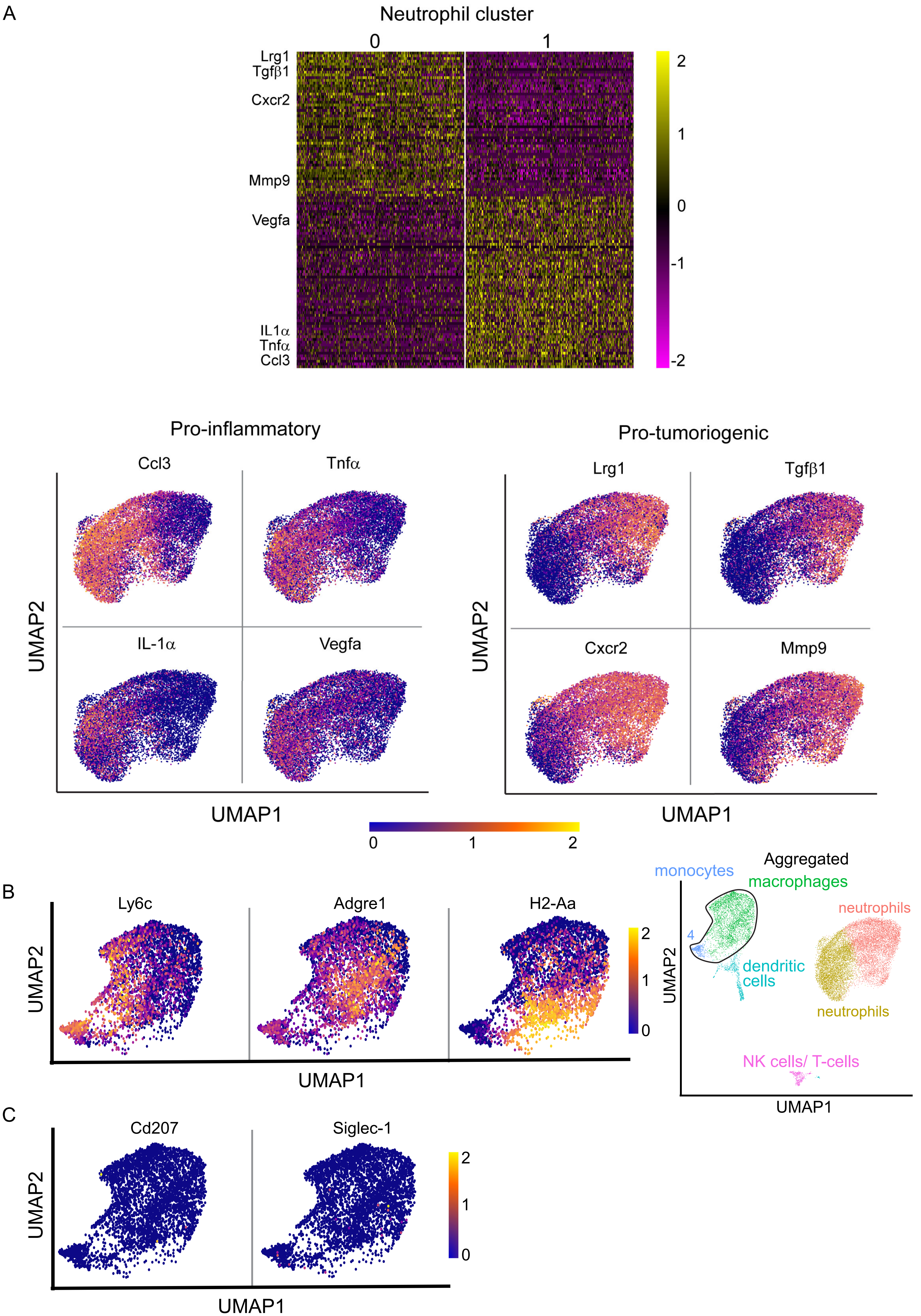
A
Neutrophil cluster
0
1
Lrg1
Tgfβ1
Cxcr2
Mmp9
Vegfa
IL1α
Tnfα
Ccl3
2
1
0
-1
-2
Pro-inflammatory
Ccl3
Tnfα
IL-1α
Vegfa
UMAP2
UMAP1
Pro-tumoriogenic
Lrg1
Tgfβ1
Cxcr2
Mmp9
UMAP2
UMAP1
0
1
2
B
Ly6c
Adgre1
H2-Aa
UMAP2
UMAP1
2
1
0
Aggregated
monocytes
macrophages
neutrophils
4
dendritic cells
neutrophils
NK cells/ T-cells
UMAP2
UMAP1
C
Cd207
Siglec-1
UMAP2
UMAP1
2
1
0

Wee et al., Figure S6

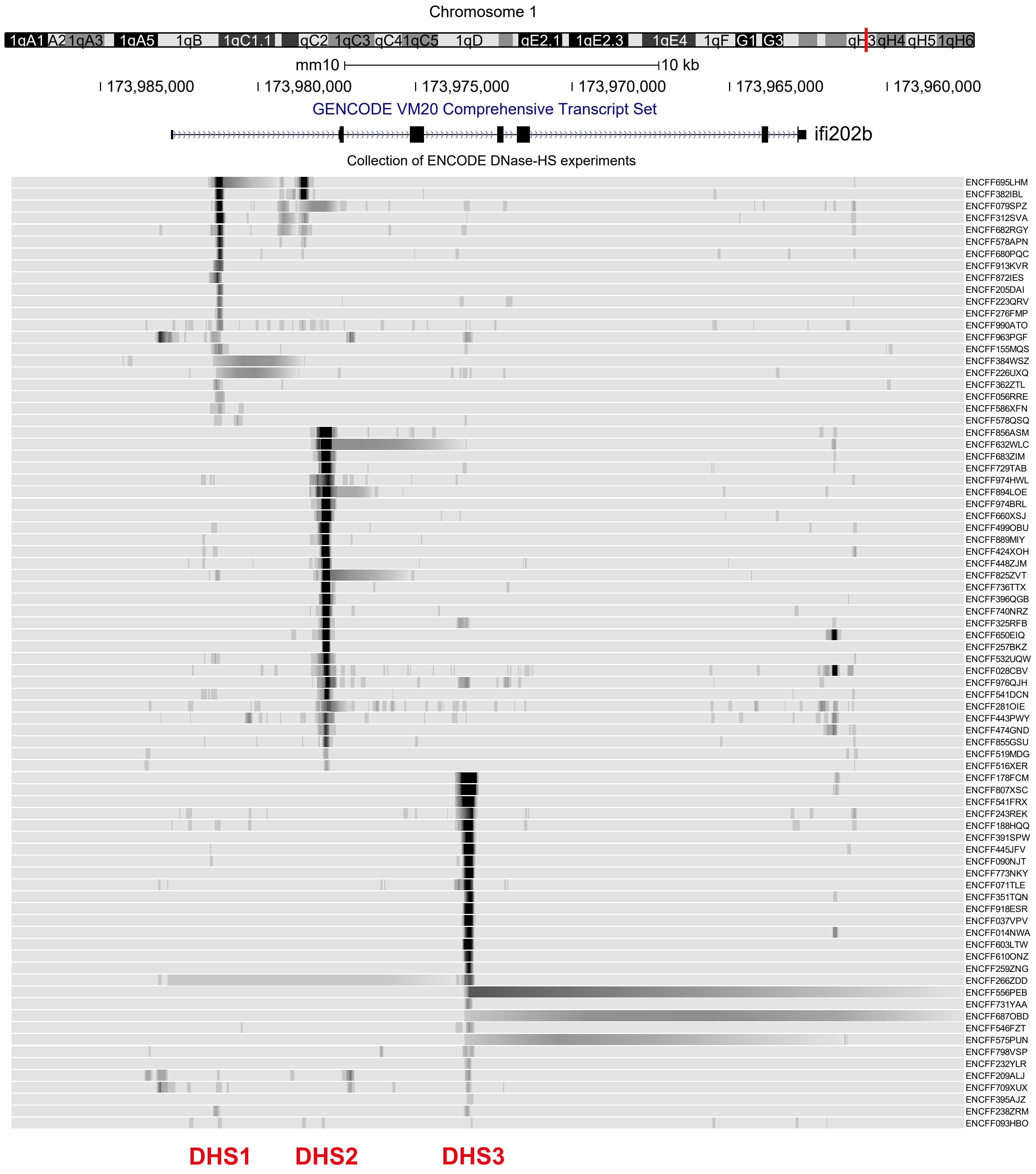

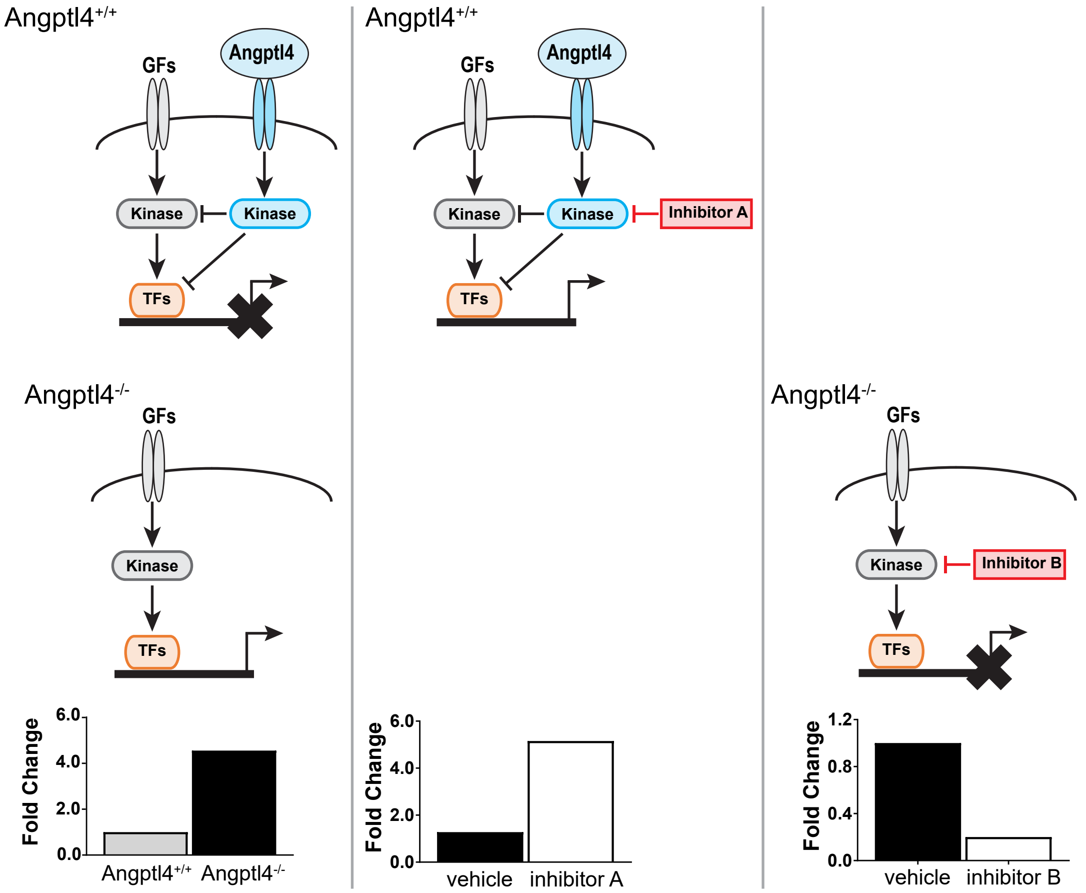

Angptl4+/+
GFs
Angptl4
Kinase
Kinase
TFs
Angptl4+/+
GFs
Angptl4
Kinase
Kinase
Inhibitor A
TFs
Angptl4-/-
GFs
Kinase
TFs
Angptl4-/-
GFs
Kinase
Inhibitor B
TFs
Fold Change
6.0
4.0
2.0
0.0
Angptl4+/+
Angptl4-/-
Fold Change
6.0
4.0
2.0
0.0
vehicle
inhibitor A
Fold Change
1.2
0.8
0.4
0.0
vehicle
inhibitor B

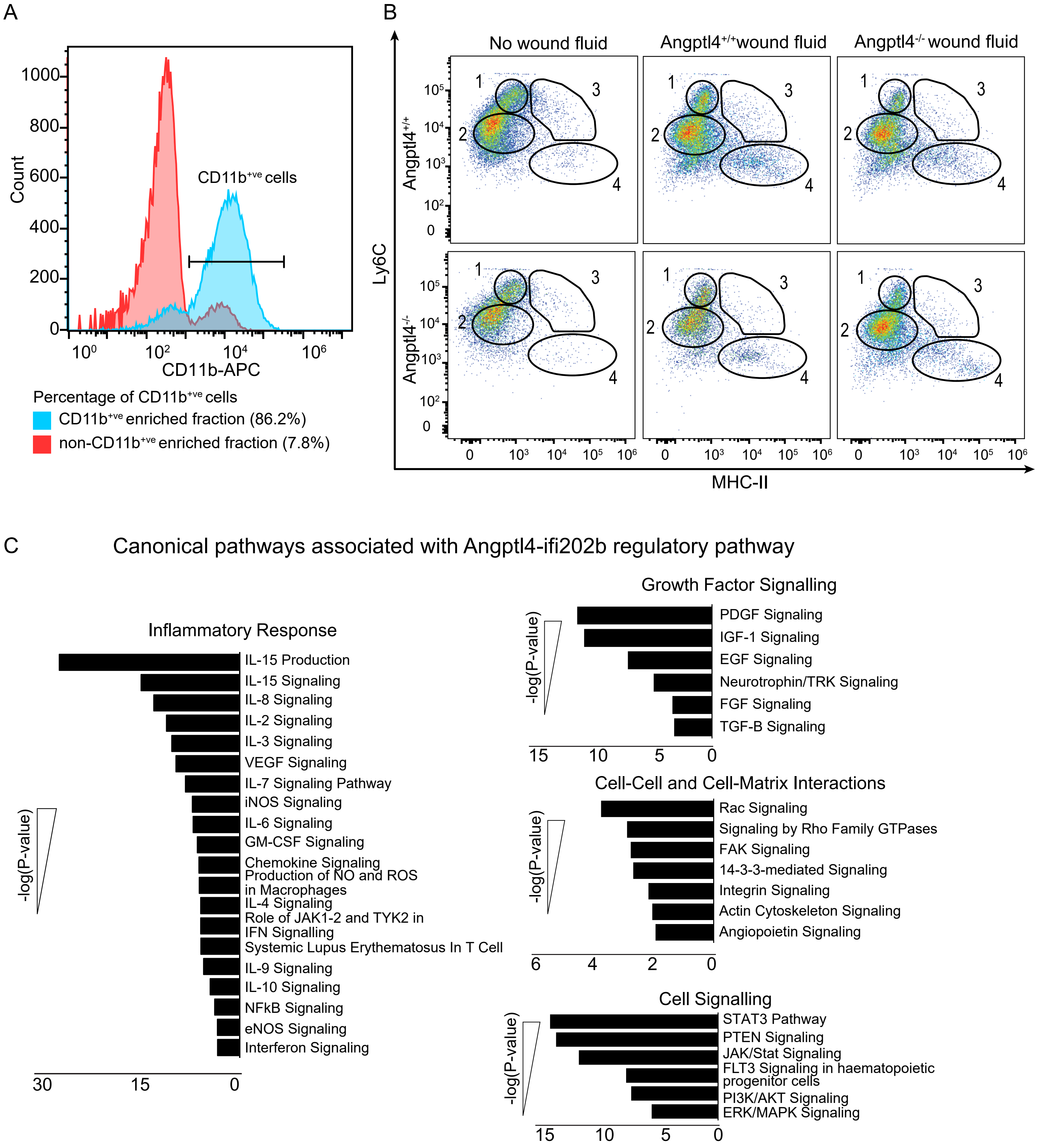
A
Count
CD11b-APC
CD11b+ve cells
Percentage of CD11b+ve cells
CD11b+ve enriched fraction (86.2%)
non-CD11b+ve enriched fraction (7.8%)
B
No wound fluid
Angptl4+/+ wound fluid
Angptl4-/- wound fluid
Angptl4+/+
Angptl4-/-
Ly6C
MHC-II
C
Canonical pathways associated with Angptl4-ifi202b regulatory pathway
Inflammatory Response
IL-15 Production
IL-15 Signaling
IL-8 Signaling
IL-2 Signaling
IL-3 Signaling
VEGF Signaling
IL-7 Signaling Pathway
iNOS Signaling
IL-6 Signaling
GM-CSF Signaling
Chemokine Signaling
Production of NO and ROS in Macrophages
IL-4 Signaling
Role of JAK1-2 and TYK2 in IFN Signalling
Systemic Lupus Erythematosus In T Cell
IL-9 Signaling
IL-10 Signaling
NFkB Signaling
eNOS Signaling
Interferon Signaling
-log(P-value)
Growth Factor Signalling
PDGF Signaling
IGF-1 Signaling
EGF Signaling
Neurotrophin/TRK Signaling
FGF Signaling
TGF-B Signaling
Cell-Cell and Cell-Matrix Interactions
Rac Signaling
Signaling by Rho Family GTPases
FAK Signaling
14-3-3-mediated Signaling
Integrin Signaling
Actin Cytoskeleton Signaling
Angiopoietin Signaling
Cell Signalling
STAT3 Pathway
PTEN Signaling
JAK/Stat Signaling
FLT3 Signaling in haematopoietic progenitor cells
PI3K/AKT Signaling
ERK/MAPK Signaling
