# Supplementary figures and images for: Single-cell analysis of skin immune cells reveals an Angptl4-ifi20b axis that regulates monocyte differentiation during wound healing
Source: Cell Death Dis. 2022 Feb 24;13(2):180. doi: 10.1038/s41419-022-04638-7 (PMC8873364; doi:10.1038/s41419-022-04638-7)

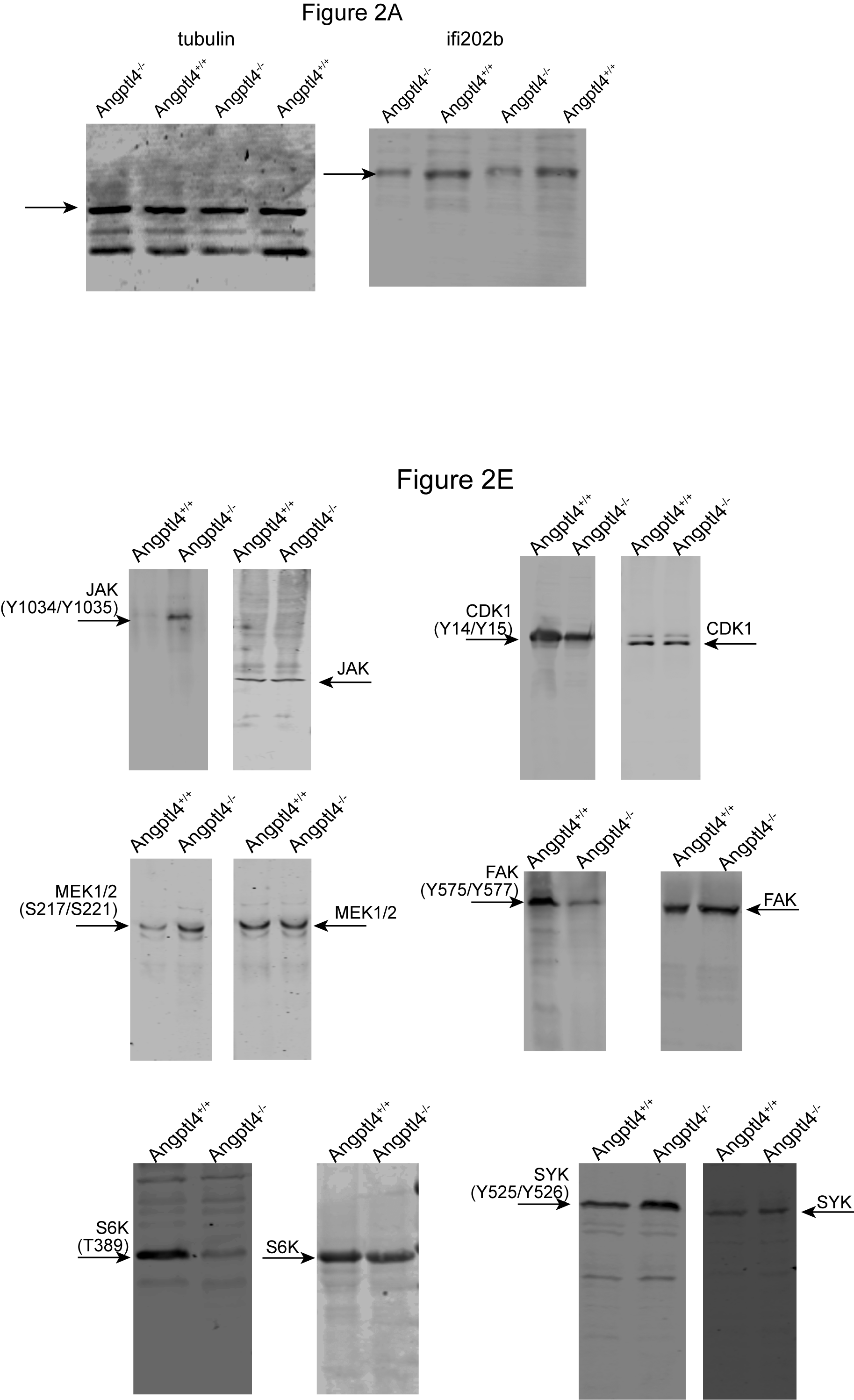

Supplement: Supplementary file 2 — RAW Westerns [file 41419_2022_4638_MOESM2_ESM.tif]
